# Dose-response-relationship of stabilisation exercises in patients with chronic non-specific low back pain: a systematic review with meta-regression

**DOI:** 10.1038/s41598-020-73954-9

**Published:** 2020-10-09

**Authors:** Juliane Mueller, Daniel Niederer

**Affiliations:** 1 Department of Computer Science | Therapy Sciences, Professorship for Physiotherapy: Exercise Science and Applied Biomechanics, Trier University of Applied Sciences, Trier, Germany; 2 Department of Sports Medicine and Exercise Physiology, Goethe University Frankfurt, Frankfurt am Main, Germany

**Keywords:** Therapeutics, Pain management, Rehabilitation

## Abstract

Stabilization exercise (SE) is evident for the management of chronic non-specific low back pain (LBP). The optimal dose-response-relationship for the utmost treatment success is, thus, still unknown. The purpose is to systematically review the dose-response-relationship of stabilisation exercises on pain and disability in patients with chronic non-specific LBP. A systematic review with meta-regression was conducted (Pubmed, Web of Knowledge, Cochrane). Eligibility criteria were RCTs on patients with chronic non-specific LBP, written in English/German and adopting a longitudinal core-specific/stabilising/motor control exercise intervention with at least one outcome for pain intensity and/or disability. Meta-regressions (dependent variable = effect sizes (Cohens d) of the interventions (for pain and for disability), independent variable = training characteristics (duration, frequency, time per session)), and controlled for (low) study quality (PEDro) and (low) sample sizes (n) were conducted to reveal the optimal dose required for therapy success. From the 3,415 studies initially selected, 50 studies (n = 2,786 LBP patients) were included. N = 1,239 patients received SE. Training duration was 7.0 ± 3.3 weeks, training frequency was 3.1 ± 1.8 sessions per week with a mean training time of 44.6 ± 18.0 min per session. The meta-regressions’ mean effect size was d = 1.80 (pain) and d = 1.70 (disability). Total R^2^ was 0.445 and 0.17. Moderate quality evidence (R^2^ = 0.231) revealed that a training duration of 20 to 30 min elicited the largest effect (both in pain and disability, logarithmic association). Low quality evidence (R^2^ = 0.125) revealed that training 3 to 5 times per week led to the largest effect of SE in patients with chronic non-specific LBP (inverted U-shaped association). In patients with non-specific chronic LBP, stabilization exercise with a training frequency of 3 to 5 times per week (Grade C) and a training time of 20 to 30 min per session (Grade A) elicited the largest effect on pain and disability.

## Introduction

Exercise is evident for the management of chronic, non-specific low back pain in therapy and rehabilitation^1–4^. In general, strength/resistance and coordination/stabilisation exercise programmes appear to be superior to other interventions in the treatment of chronic low back pain^5^. Specifically, the effects of motor control exercise therapies on the reduction of pain and disability, as well as on improvements in functional performance, are highlighted in numerous meta-analyses on chronic, non-specific low back pain, as an acute, long term^2^, and sustainable treatment^6^. These types of sensorimotor/stabilisation training are the most established therapy forms in low back pain treatment which aim to improve neuromuscular deficits^2,5^. The use of the following interventions indicate the sensorimotor training principles in the context of chronic, low back pain treatment: motor control, sensorimotor, perturbation, neuromuscular, core stability, stabilisation, Pilates-based stabilisation and instability training. The superordinate principle, musculoskeletal control by afferent sensory/proprioceptive input, central nervous system integration of the afferences and optimal stabilisation to ensure functional dynamic joint stability during perturbative situations, are key components of all the above mentioned training forms^7^. The meta-analyses on the effects of these training forms^2–4,8,9^ have not pointed out training characteristics (period, duration, frequency, intensity, etc.) for the likely largest effect. The optimal dose for the maximal treatment success-response relationship is, thus, still unknown^1,10^.

It is evident that the success of exercise interventions in the therapy of musculoskeletal disease (including non-specific low back pain) is dependent on the high adherence of the patients to their therapy plan. Regarding the therapy of chronic, non-specific low back pain, the dose-response relationship between stabilisation exercise interventions and pain reduction is of great interest to policy makers, clinicians and individuals. van Tulder et al.^4^ reported in their systematic review that a high training dosage (≥ 20 h) is more effective in exercise interventions to improve pain and function in chronic, non-specific low back pain patients. More information on the period, duration, frequency and intensity were not presented. Saragiotto et al.^2^ reported a wide range in the duration of the applied motor control intervention programmes in the studies included in their meta-analysis of 20 days to 12 weeks. The number of treatment sessions per week ranged from one to five sessions. Consequently, as a result of, inter alia, this variance in training scheduling, a large heterogenity was found in the meta-analyses highlighted above. Decreasing this heterogeneity would, on the one hand, increase the level of evidence of the stabilisation exercises’ effects on low back pain patients. On the other hand, with a much higher impact on clinical and scientific practice, the determination of an optimal dose-response relationship with the thereof derived recommendations on how an intervention needs to be structured in terms of training type, duration, frequency and intensity, is of great relevance. As an impact of a high risk of bias^11^ and a low sample size^12^ of the studies included into meta-analyses is known, these potential confounders should be considered in dose-response-analyses, likewise.

The purpose of this systematic review with meta-regressions was to (1) delineate the dose-response-relationship of stabilisation exercises and (2) derive recommendations for the stabilisation exercises’ training specifics that could maximise the reduction of pain and disability in chronic, non-specific low back pain patients.

## Methods

The presented systematic review with meta-regression was conducted in accordance with the recommendations of the Preferred Reporting Items for Systematic Reviews and Meta-Analysis (PRISMA)^13^.

### Literature research

The literature research was performed using the digital peer review-based databases PubMed (Medline), Web of Knowledge and the Cochrane Library. The following Boolean search syntax was applied (example for the PubMed-search): (stabili* OR sensorimotor OR “motor control” OR neuromuscular OR perturbation) AND (exercise OR training OR therapy OR intervention OR treatment) AND ("low back pain" OR lumbalgia OR "lower back pain" OR dorsalgia OR backache OR lumbago OR LBP OR “back pain”).

Two reviewers (JM & DN) independently conducted the literature research. Consequently, the identified studies were screened for eligibility, using firstly the titles and secondly the abstracts. Afterwards, the remaining full texts were assessed for eligibility by applying the inclusion and exclusion criteria (Table 1). A consensus was used to address any disparities; a third reviewer (N.N.) was planned to be asked, if necessary, to address any disparities. After study retrieval, additional studies were identified by manually searching through the reference list (cross-referencing) of the selected articles. The search was limited to full-text availability, publication up to the 30th of March 2020 and in the languages of English or German (Table 1).^6^

**Table 1:** Inclusion and exclusion criteria for both the studies and participants.

Criterion	Inclusion	Exclusion
Study design	Randomised controlled	Case studies, case–control, controlled, cohort studies, reviews (e.g. with meta-analysis), protocols, non-controlled intervention studies
Population	Adults	< 18 years of age
	Low back pain patients	
	Pain duration: Sub-acute, chronic, chronic-recurrent	
Intervention	Motor control exercise Core-specific sensorimotor/neuromuscular/sensorimotor/perturbation/core stability/stabiliz(s)ation/stabiliz(s)ation exercises/training	Static (non-dynamic) (motor control) exercises
	Duration of at least 2 weeks	
Control/Comparator	Active (any type of exercise, stretching, general strengthening)	
	Passive comparators (e.g. manual therapy)	
	Advice to stay active, Usual care	
	Real control (inactive, waiting control)	
Outcome	At least one measure of pain (e.g. VAS, NRS, Korff) and/or disability (e.g. ODI, RMDQ, KORFF)	
	Outcome assessment at baseline and at least once at 2 week to 24 week post-intervention-initiation	
Other	Publication or e-pub before 30th March 2020	
	Language: German & English	
	Full-text availability	

### Inclusion and exclusion criteria

The inclusion and exclusion criteria were defined with respect to population, intervention, control/comparator and outcome (PICO). The detailed criteria for both the participants and studies are displayed in Table 1.

### Data extraction

The common effect estimators for pain intensity and disability were retrieved from each study. The intervention group baseline-to-post effects sizes (Cohens d) were calculated as the change in mean values from baseline to post intervention assessment divided by the baseline standard deviation values for the respective scale. All data of interest were retrieved from the individual study data; for this purpose, a data extraction form designed for this review was used. Data on training dose and frequency were retrieved according to the TIDieR checklist. One researcher recorded all the pertinent data from the included articles and the other author independently reviewed the extracted data for its relevance, accuracy and comprehensiveness. A consensus was used to address any disparities; a third reviewer (N.N.) was asked, if necessary, to address any disparities. Authors of those studies included in this review who had not reported sufficient details in the published manuscript, were personally addressed by e-mail requesting the provision of further data. The effect estimators for pain intensity and disability were calculated using either the visual analogue scale (VAS), the numeric rating scale (NRS) or the sum score, inherent of the scale/assessment tool (0–10, 0–24 or 0–100), as the calculation of the standard mean differences is scale independent. For such data, only the direction (lower values mean less pain, less disability) was normalised. For scale-dependent calculations (inverse weighting, calculated as sample size divided by the squared standard deviation of the baseline-to-post difference), z-transformed (0–10) variables were used. Missing standard deviations for the differences were imputed according to the procedure described by Follmann et al.^14^.

### Study quality assessment

The Physiotherapy Evidence Database (PEDro; 11 criteria) scale was used to assess the methodological quality of all trials included. The PEDro scale is a valid and reliable tool to rate the internal study validity and methodological quality of controlled studies^15^. If available, the validated rating scores of the articles were taken directly from the PEDro database (website; 35 out of 46 articles). If not, both authors evaluated the articles, each criterion was rated as 1 (definitely yes) or 0 (unclear or no); potential disagreements were discussed between the two authors and resolved. Overall, the scale ranges from 0 (high risk of bias) to 10 (low risk of bias) with a sum score of ≥ 6 representing a cut-off score for studies with a sufficient study quality. As study quality was considered as a potential explanator of the effect size homogeneity, all studies, irrespective of the quality, were analysed.

### Risk of bias within the studies

The two review authors (JM and DN) independently rated the risk of bias of the outcomes pain and disability in the included studies by using the Cochrane Collaboration’s tool Risk of Bias tool 2^16,17^ . Studies’ outcomes were graded for risk of bias in each of the following domains: sequence generation, allocation concealment, blinding (participants, personnel, and outcome assessment), incomplete outcome data, selective outcome reporting and other sources of bias. For the outcomes, each item was rated as “high risk”, “low risk” or “unclear risk” of bias. Again, any disagreements were discussed between the raters. If a decision could not be reached after discussion, a third reviewer (N.N.), was included to resolve any conflicts. As the risk of bias was (indirectly, via the PEDro sum score) considered as a potential explanator of the effect size homogeneity, all studies, irrespective of the risk of bias, were analysed in the meta-regressions.

### Risk of bias across the studies

The calculation of the risk of publication bias across all the studies was indicated by using funnel plots/graphs^18^. The Review Manager 5.3 (RevMan, Version 5.3, Copenhagen: The Nordic Cochrane Centre, The Cochrane Collaboration, 2014) was used for funnel plotting.

### Data processing and statistical analysis

Data was initially plotted using scatterplot diagrams. The type of association between each independent and dependent variable was visually determined. In case of a linear association, data were processed as real values, thus, if a curve-linear association was determined, data were re-calculated using logarithmic transformations (log-association) and, respectively, Taylor-series (U-shaped-associations) to provide linearity for the regression calculation.

Sensitivity meta-regressions for dose-response analyses and the impact of study quality were conducted as described in Niederer & Mueller (2020)^6^. A syntax for SPSS (IBM SPSS 23; IBM, USA) was used (David B. Wilson; Meta-Analysis Modified Weighted Multiple Regression; MATRIX procedure Version 2005.05.23). Inverse variance weighted regression models with random intercepts (random effect model, fixed slopes model) with the dependent variables of pain intensity and disability effects (simple pre-post Cohen’s ds) and the independent variables: intervention duration [weeks, U-shaped], intervention frequency [number of trainings/week, U-shaped], intervention duration [minutes, logarithmised], intervention total dose [minutes] were applied. The sample size (SE group) and the study quality PEDro sum score [points, linear] were considered as co-factors. Homogeneity analysis (Q- and p-values) and meta-regression partial coefficients B (95% confidence intervals and p-values) were calculated. All statistical analyses were tested against a 5% alpha-error probability level.

### Effect estimators’ level of evidence

The quality of the evidence revealed by the meta-analyses was graded using the tool established by the GRADE working group^19^. Quality evidence was categorised as “very low” (The estimate of effect is very uncertain), “low” (further research is likely to change the estimate), “moderate” (further research may change the estimate) or “high” (further research is very unlikely to change the estimate of effect) (plus interim values). The grading starts with the type of evidence (RCT = high, Observational = low, all other study types = very low) and is decreased or increased based on study limitations, inconsistencies, uncertainty about directness, imprecise data, reporting bias (decreasing items), or strong associations, dose-response findings, and confounder plausibility (increasing items)^19^.

Recommendations were derived using a clinical guideline developing tool^20^. Overall, four key factors were applied to determine the strength of the recommendations: Balance between desirable and undesirable effects (larger differences between desirable undesirable effects lead to stronger recommendations)—Quality of the available evidence—Values and preferences (higher variations lead to weaker recommendations)—costs (higher costs lead to weaker recommendations. Details that are more comprehensive can be found in^21^.

## Results

### Study selection

**Figure 1:**
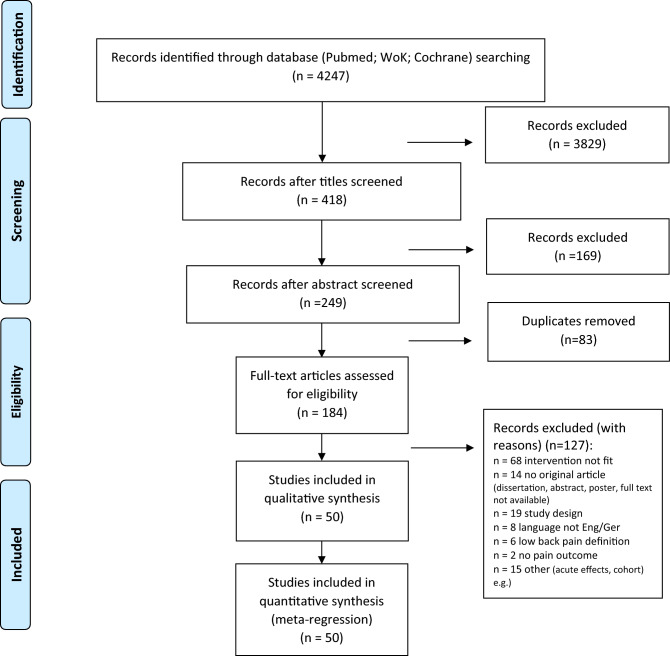
Research, selection and synthesis of included studies. n, number; Eng, English; Ger, German; WoK, web of knowledge.

The database search was completed in 03/2020. Figure 1 displays the research procedure and the flow of the study selection and inclusion.

### Study characteristics and individual studies’ results

Fifty (50) studies were included in the qualitative and in the quantitative analyses. Study characteristics and the main results are displayed in Table 2. For each of the studies included, methodological aspects, participants’ characteristics and key results are presented. Overall, 2,786 participants, thereof n = 1,239 stabilisation exercise participants, were included in the analysis.

**Table 2:** Study quality (Pedro scale) and risk of bias assessment.

Item/Study	Pedro												Risk of bias assessment						
	1	2	3	4	5	6	7	8	9	10	11	Sum PEDro	Random sequence generation	Allocation concealment	Blinding of participants and personnel*	Blinding of outcome assessment*	Incomplete outcome data	Selective reporting	Other bias
Alp, 2014	1	1	0	1	0	0	1	1	0	1	1	6	Low	High	High	Low	Unknown	Low	Low
Alrwaily, 2019	1	1	1	1	0	0	0	1	0	1	1	6	Low	Low	High	High	Unknown	Low	Unknown
Andrusaitis, 2011	0	1	1	1	0	0	1	1	0	1	1	7	Low	Low	High	Low	Unknown	Low	High
Arampatzis, 2017	1	1	0	1	0	0	0	1	0	1	1	5	Low	High	High	High	Unknown	Low	Low
Areeudomwong, 2019	1	1	1	1	0	0	1	1	1	1	1	8	Low	Low	High	Low	Low	Low	Unknown
Bae, 2018	1	1	0	1	0	0	0	1	0	1	1	5	Unknown	High	High	High	Unknown	Low	Low
Bauer, 2019	1	1	1	1	0	0	1	0	1	1	1	7	Low	Low	High	Low	High	Low	Low
Brooks 2012	0	1	0	1	1	0	1	1	1	1	1	8	Unknown	High	High	Low	Low	Unknown	Unknown
Chung, 2018	1	1	0	1	0	0	0	1	0	1	1	5	Unknown	High	High	High	Unknown	Unknown	Unknown
Critchley, 2007	1	1	1	1	0	0	1	0	1	1	1	7	Low	Low	High	Low	High	Low	Unknown
Da Luz, 2019	1	1	1	1	0	0	1	1	1	1	1	8	Low	Low	High	Low	Low	Low	Low
Demirel, 2019	0	1	1	1	0	0	0	1	0	1	1	6	Low	Low	High	High	Unknown	Low	Unknown
Ferreira 2007	1	1	1	1	0	0	1	1	1	1	1	8	Low	Low	High	Low	Low	Low	Low
Franca 2012	1	1	1	1	0	0	1	1	1	1	1	8	Unknown	Low	High	Low	Low	Low	Low
Ghorbanpour, 2018	0	1	0	1	0	0	0	1	0	1	1	5	Low	High	High	High	Unknown	Low	Low
Hosseinifar, 2013	1	1	0	1	0	0	1	0	0	1	1	5	Low	High	High	Low	High	Low	Unknown
Hwang, 2013	1	1	0	1	0	0	0	0	0	1	1	4	Unknown	High	High	High	High	Low	High
Ibrahim, 2018	1	1	1	1	0	0	1	1	1	1	1	8	Low	Low	High	Low	Low	Low	Low
Inani, 2013	1	1	0	1	0	0	0	1	0	1	1	5	Unknown	High	High	High	Unknown	Unknown	High
Khodadad, 2019	1	1	1	1	0	0	1	1	1	1	1	8	Low	Low	High	Low	Low	Low	Unknown
Kim, 2018	1	1	0	1	0	0	0	0	1	1	1	5	Low	High	High	High	High	High	Unknown
Kim, 2019	1	1	0	1	0	0	0	0	1	1	1	5	Low	High	High	High	High	Low	Unknown
Ko, 2018	0	1	0	1	0	0	0	0	0	1	1	4	Unknown	High	High	High	High	Low	High
Kofotolis, 2016	1	1	1	1	0	0	0	0	0	1	1	5	Unknown	Low	High	High	High	Low	Low
Lee, 2014	1	1	0	1	0	0	0	1	0	1	1	5	Low	High	High	High	Unknown	Unknown	High
Lee, 2011	0	1	0	1	0	0	0	0	0	1	1	4	Unknown	High	High	High	High	Unknown	High
Letafatkar, 2017	0	1	0	1	0	0	0	0	0	1	1	4	Unknown	High	High	High	High	Low	Unknown
Liu, 2019	1	1	0	1	0	0	0	1	0	1	1	5	Low	High	High	High	Unknown	Low	Unknown
Lomond, 2015	1	1	0	1	0	0	1	1	0	1	0	5	Unknown	High	High	Low	Unknown	Low	Low
Macedo, 2012	1	1	1	1	0	0	1	1	1	1	1	8	Low	Low	High	Low	Low	Low	Low
Marshall, 2013	1	1	1	1	1	0	1	1	1	1	1	9	Unknown	Low	High	Low	Low	Low	Unknown
Miller, 2013	1	1	0	1	0	0	0	1	0	1	1	5	Unknown	High	High	High	Unknown	Low	Unknown
Moon, 2013	1	1	0	1	0	0	1	1	0	1	1	6	Unknown	High	High	Low	Unknown	Low	Low
Noormohammadpour, 2018	1	1	1	1	0	0	1	0	1	1	1	7	Unknown	Low	High	Low	High	Low	Low
Rabin, 2014	1	1	1	1	0	0	0	0	1	1	1	6	Unknown	Low	High	High	High	Low	Unknown
Rasmussen-Barr, 2003	1	1	0	1	0	0	0	1	0	1	1	5	Unknown	High	High	High	Unknown	Low	Unknown
Rasmussen-Barr, 2009	1	1	1	1	0	0	0	1	1	1	1	7	Low	Low	High	High	Low	Low	Low
Rhee, 2012	1	1	1	1	0	0	0	0	0	1	1	5	Low	Low	High	High	High	Low	Unknown
Salamat, 2017	1	1	0	1	0	0	0	0	0	1	1	4	Unknown	High	High	High	High	Low	Unknown
Seo, 2019	0	1	0	1	0	0	0	0	0	1	1	4	Low	High	High	High	High	Low	Unknown
Shamsi 2017	0	1	0	1	0	0	0	1	0	1	1	5	Low	High	High	High	Unknown	Low	High
Shaughnessy, 2004	1	1	0	1	0	0	0	1	0	1	1	5	Unknown	High	High	High	Unknown	Low	Unknown
Soundararajan, 2016	0	1	0	1	0	0	0	0	0	1	1	4	Unknown	High	High	High	High	Unknown	Low
Sung, 2013	1	1	1	1	0	0	1	0	0	1	1	6	Unknown	Low	High	Low	High	Low	Unknown
Ulger, 2017	1	1	0	1	0	0	1	0	0	1	1	5	Unknown	High	High	Low	High	Low	Unknown
Unsgaard-Tondel, 2010	1	1	1	1	0	0	0	1	1	1	1	7	Low	Low	High	High	Low	Low	Low
Vikranth, 2015	1	1	0	1	0	0	0	1	0	1	1	5	Unknown	High	High	High	Unknown	Unknown	Unknown
Waseem, 2018	1	1	1	0	0	0	0	1	0	1	0	4	Unknown	Low	High	High	Unknown	High	Unknown
Woo, 2016	0	1	0	1	0	0	0	1	0	1	1	5	Unknown	High	High	High	Unknown	High	High
Young, 2015	1	1	0	1	0	0	0	0	0	0	1	3	Unknown	High	High	High	High	High	High

All included studies adopted a randomised controlled design (RCT). The main inclusion criterion was (chronic) non-specific low back pain ≥ 4 weeks^22^, ≥ 6 weeks^23^, ≥ 7 weeks^24^, ≥ 8 weeks^25–27^, ≥ 12 weeks^28–55^, ≥ 24 weeks^56–58^ and ≥ 2 year history^59^, whilst in 11^60–70^ studies this information was not presented. The baseline pain, effect sizes (Cohen’s d, stabilisation exercise group only) for pain and disability are presented in Table 3.

**Table 3:** Study characteristics (left columns) and the individual studies’ results (right columns). For each of the studies included, the methodological aspects, participants’ characteristics and key results are displayed.

Study information			Population					Assessments	Outcomes	
First author, year	Citation number	Study design, no of study arms	Main inclusion criterion LBP (time, other)	N (Total, per grop) (SE, C, C2…))	Age Mean ± SD (years)	Sex (f/m)	Baseline-pain (Scale, mean, SD if not stated otherwise)	Measurement time points total (N: weeks (if not, stated otherwise) after Baseline)	Primary outcome pain, scale, Co-hens d, (M0-M1)	Primary outcome disability name, Cohens d,, (M0-M1)
Alp, 2014	^56^	RCT, 2 SE Ctrl	CLBP ≥ 24 weeks	48, 24, 24	25–64, 48, 51	48/0	VAS (0–10), 6, range 4–9 6, range 1–10	2: 0; 12	VAS (0–10) SE: 0.8 Imputed from Saragiotto et al.^2^	RMDQ SE: 0.59 Imputed from Saragiotto et al.^2^
Alrwaily, 2019	^28^	RCT, 2 SE Ctrl	CLBP ≥ 12 weeks, NPRS ≥ 3 MODQ score ≥ 20%	30 15, 15	38.3 ± 11.3, 33.4 ± 9.0	19/11 11/4 8/7	4.4 ± 1.8 4.2 ± 1.9	2: 0; 6	NPRS (0–10) SE: 1.29	MODQ SE: 1.76
Andrusaitis, 2011	^60^	RCT, 2 SE Ctrl	nonspecific, CLBP	10, 5, 5	Range: 30–55	10/0 5/0 5/0	VAS (0–10), 4.83, range 4.3–5.5, 5.08, range 0.5–7.7	2: 0; 7	VAS (0–10) SE: 1.60	ODI SE: 1.68
Arampatzis, 2017	^29^	RCT, 2 SE Ctrl	LBP ≥ 12 weeks	40, 20, 20	31.9 ± 6.0, 31.4 ± 5.5	N.A	VAS (0–10), 3.96 ± 1.41, 4.22 ± 1.66	2: 0; 13	VAS (0–10) SE: 0.60	N.A
Areeudomwong, 2019	^30^	RCT, 3 SE Ctrl 1 Ctrl 2	CLBP ≥ 12 weeks	45 15 15 15	24.08 ± 1.00 24.00 ± 8.47 24.36 ± 9.97	34/11 11/4 12/3 11/4	4.40 ± 1.40 4.13 ± 0.92 4.07 ± 1.28	3: 0; 4; 12	NRS (0–10) SE: 2.61	Functional disability SE: 1.44
Bae, 2018	^31^	RCT, 2 SE Ctrl	LBP ≥ 12 weeks	36, 18, 18	32.7 ± 6.1, 32.4 ± 11.0	18/20	VAS (0–10), 2.9 ± 0.8, 3.0 ± 1.3	4: 0; 4; 8; 16	VAS (0–10) SE: 1.0	ODI SE: 0.19
Bauer, 2019	^22^	RCT, 2 SE Ctrl	LBP ≥ 4 weeks NRS ≥ 3	83 42 41	45.7 ± 7.8 46.7 ± 7.7	83/0	34.0 ± 21.0 28.0 ± 21.1	3: 0; 24; 48	VAS (0–100) SE: 0.42	N.A
Brooks, 2012	^32^	RCT, 2 SE Ctrl	LBP ≥ 12 weeks	64, 32, 32	36.2 ± 8.2, 36.3 ± 6.3	40/24	VAS (0–10) 3.6 ± 2.1, 4.5 ± 2.5	2: 0; 8	VAS (0–10) SE: 0.58	ODI SE: 1.08
Chung, 2018	^61^	RCT, 2 SE I SE II	CLBP	27, 14, 13	32.47 ± 7.89, 34.18 ± 6.59	17/10	VAS (0–10) 6.63 ± 1.21, 6.55 ± 1.09	2: 0; 6	VAS (0–10) SE I: 4.35 SE II: 2.95	Korean Version of ODI SE I: 3.22 SE II: 1.95
Critchley, 2007	^33^	RCT, 3 SE Ctrl 1 Ctrl 2	CLBP ≥ 12 weeks	212 72 71 69	44 ± 13 45 ± 12 44 ± 12	133/89	NRS (0–100), mean, 95%CI 67, 61–73 60, 54–66 59, 52–65	4: 0; 24; 48; 72	NRS (0–100) SE: 1.08	RMDQ SE: 0.23
da Luz, 2019	^62^	RCT, 3 SE Ctrl 1 Ctrl 2	CLBP VAS ≥ 4	30 10 10 10	26.40 ± 3.41 25.50 ± 5.28 27.10 ± 4.95	30/0	6.4 ± 0.8 6.6 ± 1.1 6.8 ± 0.4	3: 0; 4; 24	VAS (0–10) SE: 5.12	ODI SE: 2.02
Demirel, 2019	^34^	RCT, 2 SE Ctrl	CLBP ≥ 12 weeks	77 37 40	45.59 ± 12.32 44.25 ± 8.71	62/15 29/8 33/7	2.62 ± 2.23 2.92 ± 2.65	2: 0; 6	VAS (0–10) SE: 0.39	ODI SE: 0.75
Ferreira, 2007	^35^	RCT, 3 SE Ctrl. 1 Ctrl. 2	LBP ≥ 12 weeks	240, 80, 80, 80	51.9 ± 15.3, 54.8 ± 15.3, 54.0 ± 14.4	165/75	VAS (0–10), 6.3 ± 2.0, 6.5 ± 2.1, 6.2 ± 2.0	4: 0; 8, 24; 48	VAS (0–10) 0.92	RMDQ SE: 1.15
Franca, 2012	^36^	RCT, 2 SE Ctrl	LBP ≥ 12 weeks	30, 15, 15	42.1 ± 8.2, 41.5 ± 4.4	N.A	VAS (0–10), 5.94 ± 1.56, 6.35 ± 1.51	2: 0; 6	VAS (0–10) SE: 3.77	ODI SE: 3.83
Ghorbanpour, 2018	^57^	RCT, 2 SE Ctrl	LBP ≥ 24 weeks	30, 15, 15	23.8 ± 3.5, 20.9 ± 1.2	16/14	VAS (0–10), 29.5 ± 4.8, 28.3 ± 6.5	2: 0; 6	VAS (0–100) SE: 0.94	Persian version of the Quebec Low Back Pain Disability Scale Questionnaire SE: 0.33
Hosseinifar, 2013	^37^	RCT, 2 SE Ctrl	LBP ≥ 12 weeks	30, 15, 15	40.1 ± 10.8, 36.6 ± 8.2	N.A	VAS (0–100), 4.33 ± 1.58, 4.40 ± 1.95	2: 0; 6	VAS (0–100) d = 1.77	FRI questionnaire d = 1.45
Hwang, 2013	^71^	RCT, 3 SE Ctrl. 1 Ctrl. 2	LBP ≥ 12 weeks	21, 7, 7, 7	45.7 ± 8.5, 44.8 ± 7.9, 45.8 ± 9.2,	10/11	VAS (0–10), N.A., 5.83 ± 0.38, 5.71 ± 0.61	2: 0; 4	VAS (0–10) SE: 3.32	ODI SE: 1.18
Ibrahim, 2018	^38^	RCT, 3 SE Ctrl 1 Ctrl 2	LBP ≥ 12 weeks	30 10 10 10	48.5 ± 14.9 50.3 ± 9.09 49.9 ± 8.82	6/25 3/7 1/9 2/8	6.00 ± 1.41 6.00 ± 1.41 6.80 ± 1.31	2: 0; 6	NPRS (0–10) SE: 2.13	ODI SE: 0.97
Inani, 2013	^63^	RCT, 2 SE Ctrl	diagnosed with non-specific LBP	30, 15, 15	27.8 ± 7.3, 32.9 ± 64	10/20	VAS (0–10), 6.3 ± 1.8, 7.0 ± 1.6	2: 0; 12	VAS (0–10) SE: 2.72	Modified ODI SE: 2.28
Khodadad, 2019	^39^	RCT, 3 SE Ctrl 1 Ctrl 2	LBP ≥ 12 weeks	52 17 17 18	42.2 ± 3.78 44.3 ± 1.43 44.4 ± 2.17		6.2 ± 1.48 5.5 ± 1.03 5.6 ± 1.45	2: 0; 8	NRS (0–10) SE: 1.89	N.A
Kim, 2018	^40^	RCT, 2 SE I SE II	LBP > 12 weeks	30 15 15	N.A 22.31 ± 1.6 22.92 ± 1.55	30/0 15/0 15/0	N.A	2: 8	N.A	ODI SE I: 1.47 SE II: 1.64
Kim, 2019	^41^	RCT, 2 Ctrl SE	LBP ≥ 12 weeks	48 24 24	N.A 26.0 ± 3.82 28.79 ± 9.05	7/15 15/11	NRS (0–10) 4.70 ± 1.04 4.73 ± 0.82	4: 4, 8, 24	NRS (0–10) SE: 3.22	ODI SE: 0.32
Ko, 2018	^64^	RCT, 3 SE Ctrl. 1 Ctrl. 2	CLBP	29, 10, 10, 9	43.1 ± 3.7, 43.6 ± 4.5, 41.3 ± 3.8	N.A	NRS (0–10), 5.5 ± 1.3, 5.3 ± 1.3, 5.2 ± 2.1	2: 0; 12	NRS (0–10) SE: 1.15	N.A
Kofotolis, 2016	^42^	RCT, 3 SE Ctrl. 1 Ctrl. 2	CLBP ≥ 12 weeks	101, 28, 37, 36	42.71 ± 6.1, 41.22 ± 8.49, 39.11 ± 8.68	101/0 37 36 28	SF-36 (bodily pain), 36.93 ± 15.52, 38.51 ± 12.62, 39.42 ± 14.49	5: 0; 4; 8; 12; 20	SF-36 pain (0–100) SE: 1.9	RMDQ SE: 0.75
Lee, 2014	^65^	RCT, 2 SE Ctrl	CLBP	40, 20, 20	34.20 ± 0.69, 34.75 ± 0.85	N.A	VAS (0–10), 7.85 ± 1.00, 7.95 ± 1.00	3: 0; 2, 4, 6	VAS (0–10) SE: 5.75	N.A
Lee, 2011	^25^	RCT, 2 SE Ctrl	LBP ≥ 8 weeks	32, 13, 19	26–63, 50.4 ± 9.1, 46.6 ± 9.1	15/17	N.A	2: 0; 4	Million pain interference visual analogue scale MVAS (0–100 mm; 15 items) SE: 0.78	N.A
Letafatkar, 2017	^66^	RCT, 2 SE Ctrl	chronic non-specific LBP; scores > 4 in RMDQ	53, 27, 26	N.A., 36.86 ± 7.16, 38.25 ± 6.19	N.A	VAS (0–10), 6.90 ± 1.87, 5.91 ± 1.31	2: 0; 5	VAS (0–10) SE: 2.9 Imputed from graph	RMDQ: SE: 2.3 Imputed from graph
Liu, 2019	^43^	RCT, 3 Ctrl SE Ctrl	LBP > 12 weeks	43 15 15 13	N.A 58.13 ± 5.38 58.4 ± 5.08 60.67 ± 2.58	35/8 12/3 12/3 11/2	VAS (0–10) 5.67 ± 0.81 5.67 ± 0.72 5.85 ± 0.89	2,:12	VAS (0–19) SE: 1.92	N.A
Lomond, 2015	^58^	RCT, 2 SE Ctrl	LBP > 24 weeks; ODI ≥ 19%	33, 12, 21	43.1 ± 11.9, 41.6 ± 10.9	15%male 6%male	NRS (0–10), 2.8 ± 1.6, 3.6 ± 1.6	2: 0; 7	NRS 0–100 SE: 1.1	ODI SE: 0.9
Macedo, 2012	^44^	RCT, 2 SE Ctrl	CLBP ≥ 12 weeks	158, 76, 82	48.7 ± 13.7, 49.6 ± 16.3	57/19 45/37	NRS (0–10), 6.1 ± 2.1, 6.1 ± 1.9	4: 0; 8, 24; 48	NRS (0–10) SE: 1.05	RMDQ: SE: 0.81
Marshall, 2013	^45^	RCT,2 SE Ctrl	Ongoing recurrent LBP ≥ 12 weeks	64, 32, 32	18–50, 36.2 ± 8.2, 36.2 ± 6.2	40/24	VAS (0–10), 3.6 ± 2.1, 4.5 ± 2.5	3: 0; 8; 24	VAS 0–10, SE: 0.9	ODI: SE: 0.93
Miller, 2013	^24^	RCT, 2 SE Ctrl	LBP ≥ 7 weeks	29, 15, 14	19–87, 54 ± 15, 44 ± 16	14/15	VAS (0–10), 4.1 ± 2.0, 3.0 ± 2.0	2: 0; 6	VAS (0–10) SE: 0.5	N.A
Moon, 2013	^46^	RCT, 2 SE I SE II	LBP ≥ 12 weeks	21, 11, 10	28.6 ± 4.9, 28.4 ± 5	7/14	VAS (0–100), 34.2 ± 17.1, 33.5 ± 18.4	2: 0; 8	VAS (0–100), SE: 0.78, SE II: 0.93	ODQ, SE: 0.84 SE II: 2.1
Noormohammadpour, 2018	^47^	RCT, 2 SE Ctrl	CLBP ≥ 12 weeks	20, 10, 10	18–55, 43.3 ± 7.5, 41.0 ± 6.4	20/0	VAS (0–100), 38.4 ± 21.7, 36.2 ± 27.2	N.A	VAS (0–100), SE: 1.6	RMDQ, SE: 2.0
Rabin, 2014	^67^	RCT, 2 SE Ctrl	CLBP	105, 48, 57	Range: 18–60	25/23, 31/26	NRS (0–10), 4,9 ± 1.7, 5.3 ± 1.7	2: 0; 8	NRS (0–10) SE: 1.5	MODI (0–100) SE: 2.0
Rasmussen-Barr, 2003	^23^	RCT, 2 SE Ctrl	LBP ≥ 6 weeks	42, 22, 20	39 ± 12, 37 ± 10	17/7 18/5	VAS (0–100), 33, 32	4: 0; 6; 12; 24	VAS (0–100) SE: 0.95 Imputed from Saragiotto et al.^2^	ODI SE: 1.18 Imputed from Saragiotto et al.^2^
Rasmussen-Barr, 2009	^26^	RCT, 2 SE Ctrl	LBP ≥ 8 weeks	71, 36, 35	36 ± 10, 40 ± 12	18/18, 18/17	VAS (0–100), 32, range 18–59, 38, range 10–47	5: 0; 8; 12; 24; 144	VAS (0–100) SE: 0.99 Imputed from Saragiotto et al.^2^	Oswestry Low Back Pain Questionnaire (OSD), n SE: 1.11 Imputed from Saragiotto et al.^2^
Rhee, 2012	^68^	RCT, 2 SE Ctrl	LBP	42, 21, 21	53.09 ± 9.04, 50.90 ± 5.24	11/10, 10/11	Million Visual VAS (0–100), 42.7 ± 13.8 32.8 ± 10.9	2: 0; 4	MVAS (0–100) SE: 0.66	ODI SE: 1.14
Salamat, 2017	^48^	RCT, 2 SE I SE II	extension related non-specific CLBP ≥ 12 weeks	24, 12, 12	35.83 ± 9.31, 36.09 ± 9.6	N.A	VAS (0–10), 5.16 ± 1.74, 5.9 ± 1.9	2: 0; 4	NRS (0–10) SE I: 1.3 SE II; 1,8	ODI SE I: 0.66 SE II: 0.76
Seo, 2019	^49^	RCT, 2 Ctrl SE	LBP ≥ 12 weeks	26 13 13	22.62 ± 1.58 22.31 ± 1.60 22.92 ± 1.55	15/11 7/6 8/5	N.A:	2: 4	N.A	ODI SE: 0.86
Shamsi, 2017	^50^	RCT, 2 SE I SE II	LBP ≥ 12 weeks, VAS 3–6	51, 27, 24	38.9 ± 12.2, 47.0 ± 9.9	33/18,	VAS (0–100), 52.4 ± 9.2, 53.0 ± 9.2	2: 0; 6	VAS (0–100), SE I: 4.0 SE II: 3.1	ODI SE I: 1.3 SE II: 1.1
Shaughnessy, 2004	^51^	RCT, 2 SE Ctrl	LBP ≥ 12 weeks	41, 20, 21	43 ± 9, 46 ± 11	27/14, 14/6, 13/8	Sf-36 (bodily pain), 31 ± 12, 32 ± 13	2: 0; 10	Sf-36 (bodily pain), SE: 0.9	ODI SE: 0.85
Soundararajan, 2016	^59^	RCT, 2 SE Ctrl	2-year history Of CLBP	30, 15, 15	26.87 ± 2.17, 27.1 ± 2.09	12/18, 6/9, 6/9	VAS (0–10), 6.27 ± 0.70, 6.6 ± 0.74	2: 0; 6	VAS (0–10) SE: 5.06	MODQ SE: 3.3
Sung, 2013	^27^	RCT, 2 SE Ctrl	Recurrent LBP ≥ 8 weeks	50, 25, 25	Range 27–63, 47.7 ± 8.9, 53.1 ± 9.1	20/30 10/15, 10/15	N.A	2: 0; 4	N.A	ODI SE: 0.26
Ulger, 2017	^52^	RCT, 2 SE Ctrl	LBP ≥ 12 weeks	113, 57, 56	Range 20–73, 41.6 ± 12.9, 43.1 ± 14.3	67/46, 35/22, 32/24	VAS (0–10), 6.69 ± 1.6, 3.0 ± 2.43	2: 0; 6	VAS (0–10) SE: 2.3	ODI SE: 1.2
Unsgaard-Tondel, 2010	^53^	RCT, 3 SE Ctrl 1 Ctrl 2	CLPB ≥ 12 weeks	109, 36, 36, 37	Range 19–60, 40.9 ± 11.5, 43.4 ± 10.2, 36.0 ± 10.3	76/33 29/7 23/13 24/13	NRS (0–10), 3.31 ± 1.42, 3.61 ± 1.75, 3.30 ± 1.74	3: 0; 8; 48	NRS (0–10) SE: 0.37	ODI SE: 0.28
Vikranth, 2015	^69^	RCT, 2 SE Ctrl	mechanical low back pain VAS < 5	30, 15, 15	Range 30–45, 37.0 ± 2.76, 37.1 ± 3.51	11/19 5/10, 6/9	VAS (0–10), 3.8 ± 0.83, 3.73 ± 1.06	2: 0; 2	VAS (0–10) SE: 0.5	ODI SE: 0.9
Waseem, 2018	^54^	RCT, 2 SE Ctrl	LBP ≥ 12 weeks	108, 53, 55	Range 20–60, 46.39 ± 7.43, 45.5 ± 6.61	37/71, 18/35, 19/36	N.A	4: 0; 2; 4; 6	N.A	ODI SE: 1.8
Woo, 2016	^55^	RCT, 2 SE I SE II	LBP ≥ 12 weeks	30, 15, 15	N.A., 39.8, 40.1	N.A	N.A	2: 0; 4	N.A	ODI, SE I: 1.85 SE II: 2.37
Young, 2015	^70^	RCT, 2 SE Ctrl	CBP	48, 24, 24	N.A	N.A	VAS (0–10), 4.3 ± 1.26, 4.0 ± 1.38	2: 0; 6	VAS (0–10) SE: 0.43	N.A

Legend: RCT, randomized controlled trial; T, total, E, exercise, SE, stabilisation exercise, Ctrl, control or comparison group; CLBP, chronic low back pain; N, number; f, female; m, male; SD, standard deviation; Mx, measurement visit number, VAS, visual analogue scale; NRS, numeric rating scale; NPRS, numeric pain rating scale; ODI, owestry disability index, RMDQ, Roland Morris disability questionnaire

### Study quality and risk of bias within studies

Both the study quality and risk of bias ratings are presented in Table 2. The overall study quality ranged from 3/10 to 9/10 points, with a mean of 5.7 ± 1.4 points on the Pedro scale.

### Individual studies' training characteristics

Table 4 summarises the individual studies’ training characteristics. All interventions and the comparators are described. The stabilisation exercises are called core stability exercise^25,27,30–32,47,51,54,61–63,69^, motor control exercise^24,35,38,44,45,48,50^, stabilisation^23,26,28,34,41,52,55,60^, lumbar stabilisation exercise^39,46,56,64,67^, spinal stabilisation^33,37,68^, sensorimotor training^66,71^, trunk stability exercise^49,58^, Swiss ball stabilisation^43,65,70^, perturbation training^29^, sling training^53^, McGill stabilisation exercise^40,57^, segmental stabilisation exercise^36^, neuromuscular exercise^22^, multifidus muscle retraining^59^ and Pilates-based exercise^42^. The intervention period ranged between 2^69^ and 24^22^ weeks with a mean of 7.0 ± 3.3 weeks. Training frequency ranged from 1^29^ to 12^53^ times per week with a mean of 3.1 ± 1.8 times; 3 studies^24, 55,63^ did not report on this information. Mean training time per session was 44.6 ± 18.0 min with a range from 15^24^ to 90 minutes^29,33^ (9 studies^35,47,49,54,62,63,65,67,68^ did not report on this aspect). The number of exercises practised per session varied between 2^35,47,49,54,62,63,65,67,68^ to 18^29^ exercises with a mean of 7.2 ± 3.9 exercises; 13 studies^30,32, 35,37,40,44,48,50,52,53,56,58^ did not report this information.

**Table 4:** Individual studies’ training specifications.

First author, year	Citation number	Type intervention (MCE, Core)	Exercises (No; Name): (Description/Name of exercises)	Type comparator(s)	Training period (weeks)	Training frequency (sessions per week) scheduled, real	Training duration (minutes per session)	Sets (number per exercise)	Repetitions (per set per exercise)	Rest (between sets per exercise; between exercises in seconds)
Alp, 2014	^56^	Lumbar core stabilization exercise (SE)	N.A	Conventional home-based exercise (HE)	6	SE: 3	45–60 (30 MCE)	SE: N.A HE: 1	SE: N.A HE: 20	N.A., N.A
Alrwaily, 2019	^28^	Stabilization exercise	5; Abdominal bracing (supine), Abdominal bracing (supine) with heel slide, Abdominal bracing (supine) with leg lifts, Abdominal bracing (supine) with bridging, Bracing with single leg bridging	Stability exercise combined with neuromuscular electrical stimulation	6	2	20	N.A	N.A	N.A.; N.A
Andrusaitis, 2011	^60^	Stabilization	2: dorsal decubitus, ventral decubitus	Strengthening	7	3	40	1	6—10	N.A.; 30 sec
Arampatzis, 2017	^29^	Perturbation-based core training	15–18: 3 different perturbation exercises in half-seated position, classical core stability exercises on unstable surfaces	No specific training, normal routine	13	2	90	3	60 sec	180—300; 120
Areeudomwong, 2019	^30^	Core stabilisation exercise	N.A.: Practiced recruitment of deep trunk muscles, particularly transversus abdominis (TrA) and lumbar multifidus (LM) muscles, together with the diaphragm and pelvic floor muscles, reducing superficial trunk muscle activity in order to improve function of deep trunk muscles and control inter-segmental lumbar spine movement during activities Exercise difficulty was increased by integrating deep muscle cocontraction with controlling movement of extremities and heavier loading positions, such as bridging, bird-dog position and single knee to chest	Proprioceptive Neuromuscular Facilitation Training; Inactive control group	4	3	30	N.A	N.A	N.A.; 60
Bae, 2018	^31^	Core stability exercises	6: Abdominal drawing-in in 4-point kneeling and supine position, Opposite upper and lower extremity lift in quadruped position, Straight leg raise exercise in prone position, Supine lower extremity extender in supine position, Straight leg raise exercise in supine position, Horizontal side-support exercise in side lying position	Assisted sit-up exercise (SUE)	4	3	30	N.A	N.A	N.A.; N.A
Bauer, 2019	^22^	Neuromuscular exercise	9; Modified curl up, Bird dog, Side bridge/Mermaid, Single leg stretch, Shoulder bridge, Weight transfer side lunge and one leg stand, “Tai chi warrior”, Lifting up an imaginary Ball, To achieve normal range of motion in thoracic region, and hip and ankle joints,	Inactive control group	24	2	60	N.A	N.A	N.A.; N.A
Brooks, 2012	^32^	Specific trunk exercise group (SEG)	N.A: Included skilled cognitive activation of the trunk muscles in addition to a number of other best practice exercises: Skilled abdominal contractions and postural training, Side lying trunk exercises (mat-based), Prone lying trunk exercises (mat-based; Hip-specific exercises, Upper and lower limb–focused exercises, Full-body exercises (reformer-based)	Seated cycling	8	3	50–60	N.A	N.A	N.A.; N.A
Chung*, 2018	^61^	Core stability exercises with flexi bar	4: Abdominal drawing-in maneuver in standing, hook-lying, quadruped, and prone positions by maintaining each motion for 10 s. It was used both hands holding the FB	No further, both groups SE	6	3	30	3	10	180; N.A:
		Core stability exercises	4: Abdominal drawing-in maneuver in standing, hook-lying, quadruped, and prone positions by maintaining each motion for 10 s	No further, both groups SE	6	3	30	3	10	180; N.A:
Critchley, 2007	^33^	Spinal stabilization (SS)	5: individual transversus abdominis and lumbar multifidus muscle training followed by group exercises that challenged spinal stability	Physio Pain Management	8	8	90	Individual	Individual	individual
Da Luz, 2019	^62^	Core stability exercise	4; prone bridge, supine bridge, side bridge, bird dog with lower limb elevation As the participants progressed throughout the program, the degree of difficulty of the exercises increased	Core stability exercise combined with neuromuscular electrical stimulation; neuromuscular electrical stimulation only	4	3	N.A	10	N.A	N.A., 60
Demirel, 2019	^34^	Stabilization exercise	4–5; The TA and multifidus muscles were contracted together with diaphragm respiration appropriately in basic positions (supine, prone, standing, sitting and crawling positions) Progress over the six weeks included different positions, use of resistance bands	Yoga exercises	6	3	60	N.A	N.A	N.A.; N.A
Ferreira, 2007	^35^	Motor control exercise	N.A.: Improving function of specific trunk muscles thought to control inter-segmental movement of the spine, including transversus abdominis, mul- tifidus, the diaphragm and pelvic floor muscles	General exercise Spinal manipulation therapy	8	12	N.A	N.A	N.A	N.A.; N.A
Franca, 2012	^36^	Segmental stabilization exercises (SSEs)	4: exercises for the TrA in 4 point kneeling, exercises for the TrA in dorsal decubitus with flexed knees, exercises for the LM in ventral decubitus, Cocontraction of the TrA and LM in the upright position	Stretching (ST)—focused on stretching the erector spinae, hamstrings, and triceps surae	6	2	30	3	15	N.A.; N.A
Ghorbanpour, 2018	^57^	McGill stabilization exercises group	3: Curl up, Side Bridge, Bird Dog with one hand or one foot and one hand and the opposite leg	Conventional physio (strengthening, stretching, flexibility)	6	3	30	3	10	N.A.; 120
Hosseinifar, 2013	^37^	Spinal stabalization seercise	N.A	McKenzie Method	6	3	60	10	N.A	N.A.; N.A
Hwang, 2013	^71^	Sensorimotor training	6: Hollowing exercise, Single leg raising in the quadruped position, contralateral arm and leg raising in the quadruped position, abdominal bracing Holding a bridging position, single leg raising in the bridging position	2 Group: 1 healthy controls ©, 1 lbp physical therapy (C LBP)	4	5	40	N.A	N.A	N.A.; N.A
Ibrahim, 2018	^38^	Motor control exercise	4–12; Abdominal drawing in in supine, in quadruped, in sitting, in standing, in supine with heel slide, in supine with leg lift (each leg), in supine with bridging, in supine with single-leg bridge, with curl-up, horizontal side support with knees bent, in quadruped with leg raise, etc	Motor control exercise plus patient education; Patient Education only	6	2	30	N.A	10	N.A.; N.A
Inani, 2013	^63^	Core stability exercies	4: Slow curl ups, sit ups, oblique plank/side bridge, bird dog	Conventional Exercise	12	N.A	N.A	N.A	N.A	N.A.; N.A
Khodadad, 2019	^39^	Lumbar Stabilization	5; Elbow-Toe, Back Bridge, Hand-Knee, Side Bridge, Curl up	Cognitive functional treatment; Inactive control group	8	3	60	N.A	N.A	N.A.; N.A
Kim, 2018*	^40^	McGill’s exercise; Sahrmann 0–5 level Exercise	N.A.; curl up, side bridge, and bird dog	No further, both groups SE	8	3	30	N.A	N.A	N.A.; N.A
		Stabilization exercise	N.A.; Pro balance trainer and dynamic air cushion training	No further, both groups SE	8	3	30	N.A	N.A	N.A.; N.A
Kim, 2019	^41^	Stabilization exercise	4: supine pelvic lift, supine and prone bridging exercise, and side-lying hip abduction	Simulated horseback riding	8	2	30	N.A	N.A	N.A.; N.A
Ko, 2018	^64^	Lumbar stabilization (LS)	8: sit up, superman, quadruped arm & leg raise, squat, lower body fixation plank, upper body fixation plank, side plank, hip bridge	2 Groups: Sling, Control	12	3	60 min (40 min MCE)	3	10	60; 60
Kofotolis, 2016	^42^	Pilates	16: Roll down, mermaid, spine stretching, pelvic curl, criss-cross, double leg stretch, hundreds, double knee folds, table top, swimming, swan, catstretch, child’s pose, hips stretch	General strengtheing/stabilisation exercise, control	8	3	60	Progressive: 2 (until week 4), then 3	Progressive: 15 (week 1–2), 20 (w 3–4), 15 (5–6), 20 (7–8)	120; 30
Lee, 2014	^65^	Ball exercise group	10: exercises on swiss ball from sitting to bridging	PNF pattern group	6	4	N.A	2	20	15; N.A
Lee, 2011	^25^	Core stability exercises	5: upper body extension in prone position, alternate arm and leg lift in quadruped position, alternate arm and leg lift in prone position, diagonal curl-up and straight curl- up in supine position, quadruped exercises, performed from an all-fours position with the arms and legs extending	Control	4	4	20	N.A	N.A	N.A.; N.A
Letafatkar, 2017	^66^	SMT-Perturbation with HUBER machine	10: upright stance, push and pull with oscillatory perturbative movements of variable amplitude and speed	Control	5	2	30–45	2–4	2–6	N.A.; 300
Liu, 2019	^43^	Core Stabilization Exercise on Swiss ball	6: Glute Bridge Pose, Single Leg Bridge, Bridge and Double Knee Flex, Single Leg Bridge and Double Knee Flex, Reverse Bridge, Reverse Bridge and Hip and Knee Flex	Chen-Style Tai Chi	12	3	60	N.A	N.A	N.A.; N.A
Lomond, 2015	^58^	Trunk stabilization	N.A.: 3 components of spinal stability	Movement System Impairment (MSI)	6	1	45–60	N.A	N.A	N.A.; N.A
Macedo, 2012	^44^	MCE	N.A	Graded activity	8	2 (4 weeks), 1 (4 weeks)	60	1	10	N.A.; N.A
Marshall, 2013	^45^	MCE, Pilates	8: Whole body stretching, Skilled abdominal contractions and postural training, side lying trunk, prone lying trunk, hip specific exercises, upper and lower limb, full body exercises, whole body stretching	Stretching and cycling	8	3	55	N.A	N.A	N.A.; N.A
Miller, 2013	^24^	Stabilzing MCE	10: Phase one: Prone, Supine, Quadruped; Phase two: Supine leg machine, Quadruped -Alternate arm lifts, Alternate leg lifts, standing; Phase three: Quadruped-Alternate arm and leg lifts, Standing with rotation, Bridging	McKenzie	6	N.A	10–15	1	10–50	N.A.; N.A
Moon*, 2013	^46^	Lumbar stabilization exercises,	16: aimed to strengthen the deep lumbar stabilizing muscles: the transversus abdominis, lumbar multifidi, and internal obliques	No further, both groups SE	8	2	60 (35 min LSE)	1	10	N.A.; 60
		Lumbar dynamic strengthening exercises	14: activated the extensor (erector spinae) and flexor (rectus abdominis) muscle groups	No further, both groups SE	8	2	60 (35 min LDSE)	1	10	N.A.; 60
Noormohammadpour, 2018	^47^	Multi-step core stability exercise	4: 2 on floor; 2 on swiss ball	Waiting list	8	3		3	10	N.A.; N.A
Rabin, 2014	^67^	Lumbar stabilization exercise	4: Quadruped, sidelying, supine, and standing positions	Manual therapy	8	Supervised: 2 × first 4 weeks; 1 × week 5–8;	N.A	N.A	N.A	N.A.; N.A
Rasmussen-Barr, 2003	^23^	Stabilizing training	6–8: motor control, supine crooked-lying, four-point kneeling, prone, sitting and standing	Manual therapy	6	1 supervised, 1 homebased	45 supervised, 10–15 unsupervised	3	15	N.A.; N.A
Rasmussen-Barr, 2009	^26^	Graded stabilizing exercise	7: supine crooked-lying, four-point kneeling, prone, sitting, standing	30-min walk every day	8	1 supervised, 1 homebased	45 supervised, 10–15 unsupervised	3	15	N.A.; N.A
Rhee, 2012	^68^	Specific localized exercises aimed at restoring the stabilizing protective function of the spinal muscles around the spinal joint	5: Upper-body extension, alternate arm and leg lift, alternate arm and leg extension on all fours, diagonal curl-up, curl-up	Advice regarding bed rest, absence from work, prescription medications, and resuming normal activity as tolerated	4	5	N.A	N.A	N.A	N.A.; N.A
Salamat*, 2017	^48^	Movement control	N.A.: The aim of the intervention was to normalize the abnormal movement patterns and postures and to relax trunk muscles. Exercises involved training to modify pain provocative postures and movement patterns in order to decrease pain while performing the task	No further, both groups SE	4	2	45	3	15–30	60 – 120; 300
		Stabilization exercise	N.A.: Exercises involved coordinated training and independent activity of deep trunk muscles including transversus abdominis and multifidus in pain-free positions and movements	No further, both groups SE	4	2	45	3	15–30	60 – 120; 300
Seo, 2019	^49^	Trunk stability exercise	16: nine movements of mat-based trunk stability exercises and seven movements of Swiss ball trunk stability exercises	Gyrotonic exercise	4	3	N.A:	N.A	N.A	N.A.; N.A
Shamsi*, 2017	^50^	MCE	N.A.: Progressive classic stabilization	No further, both groups SE	6	3	20	N.A	10	N.A.; N.A
		Core	N.A.: Exercises were performed in a lying position starting with simple movements and advancing to more difficult exercises (e.g. on a Swiss ball)	No further, both groups SE	6	3	20	N.A	10	N.A.; N.A
Shaughnessy, 2004	^51^	Core	3: Prone lying, kneeling, supine	Standard physiotherapy	10	2 (week 1–2), 1 (week 3–10)	60 week 1, else 30	N.A	Max. 10	N.A.; N.A
Soundararajan, 2016	^59^	Multifidus muscle retraining	8: Bridging, lying prone, quadruple, prone lying, leg extension, sitting, standing, shoulder flexion	Traditional back exercises (strength and stretching)	6	3	20	1	20	N.A.; 120–240
Sung, 2013	^27^	Core	5: Knee to chest for each leg in supine position, double leg knee to chest in supine position, prayer stretch on all fours, leaning forward position while sitting, lateral side stretch in standing position	Flexibility	4	1 supervised, 6 homebased	20	2	15	N.A.; N.A
Ulger, 2017	^52^	Stabilization	N.A: Increasing intensity and changing exercises once/week	Manipulation	6	3	60	3	10	N.A.; N.A
Unsgaard-Tondel, 2010	^53^	Sling	N.A: Sling	Low-load MCE (feedback) and General exercise	8	1	40	N.A	N.A	N.A.; N.A
Vikranth, 2015	^69^	Core stabilization	8: Week 1: Transversus abdominus activation, transversus abdominus marching, pelvic tilt, segmental bridge; Week 2: Fall out, modified crunch, cat stretch, back extension	MCE (passive)	2	5	35		Week 1: 8; week 2: 15	120; N.A
Waseem, 2018	^54^	Core stabilization	7: Pressure feedback core exercise, multifidus exercise, frontal and side plank exercise, pelvic floor exercises, diaphragmatic strengthening, single leg standing on foam, tandem standing with perturbation	Routine exercise	6	1 supervised, 2 homebased	N.A	N.A	N.A	N.A.; N.A
Woo*, 2016	^55^	Lumbar stabilization exercise	6: Lower extremity lifting in a bridge posture, lower extremity lift in a prone position on a ball, upper extremity lift in a prone position on a ball, moving the body forward grasping a sling in a kneeling position, lifting the buttocks with the lower extremity hooked on a sling in a supine position;	No further, both groups SE	4	N.A	40 (30 min MCE)	Group A: 4 Group B: 2	10–12	N.A.; N.A
		Lumbar stabilization exercise with thoracic extension exercise	10: Lower extremity lifting in a bridge posture, lower extremity lift in a prone position on a ball, upper extremity lift in a prone position on a ball, moving the body forward grasping a sling in a kneeling position, lifting the buttocks with the lower extremity hooked on a sling in a supine position; plus thoracic extension exercise	No further, both groups SE	4	N.A	40 (30 min MCE)	Group A: 4 Group B: 2	10–12	N.A.; N.A
Young, 2015	^70^	Swiss ball stabilization	N.A	PNF	6	3	50	N.A	N.A	N.A.; N.A

All interventions and the respective comparators are described. exercises, stabilisation exercise; N.A., not applicable.

*Both groups were included into quantitative analysis (meta-regression).

The qualitative analysis of the training volume revealed a range of 1^30,32,35,37,40,44,48,50,52,53,56,58,70^ to 10^24,44,46,59,60^ sets per exercise practiced with a mean of 3.2 ± 2.4 sets, while 28^22,25,28,30–35,38–41,43,45,49–51,53,54,56,58,63,67–71^ studies did not report any details on this aspect. In addition to this, only 23^22,25,28,30–35,38–41,43,45,49–51,53,54,56,58,63,67–71^ studies reported on the number of repetitions per set per exercise, with a range of 6^23,24,26,27,36,38,42,44,46–48,50–52,55,57,59–61,64–66,69^ to 30^66^ repetitions (mean: 13.6 ± 5.6 repetitions per set per exercise). In addition, only 12 studies^29,30,42,46,48,57,59–62,64,65^ reported on the systematic use of rests between exercises, ranging from 15^29, 30, 42,46,48,57,59–62,64,65^ to 300^65^ s (mean: 106.3 ± 86.5 s).

### Meta-regression analysis

The results of the meta-regressions are highlighted in Table 5. The total variance explanation was 44% for pain and 15% for disability. When all the other predictors were partialized, moderate quality evidence revealed that a training duration of 20 to 30 min elicits the largest impact on the effect sizes (both in pain and disability) of stabilisation exercise training in low back pain patients. Quality of evidence was downgraded due to risk of bias (− 1), downgraded due to imprecise data (wide confidence intervals, − 1), downgraded (− 1) due to (some) uncertainty about directness, and upgraded due to dose-response-relationship (+ 1), upgraded due to: confounders were considered (+ 1).

**Table 5:** Outcomes of the sensitivity meta-regressions.

	Model R^2^	Mean effect size	N effect sizes included	Homogeneity Q	B	95% CI. LL, UL	p-value
*A Pain*							
Intervention: duration [weeks]	.445	1.8	40	31	− .009	− .1, .08	.8
Intervention: frequency [N_Trainings_/week] data transformed from U-shaped association					.164	− .239, .567	.4
Intervention: Time per session [minutes] Data transfomed from negative log association					− 1.75	− 2.61, − .879	.0001
PEDro sum score [points]					− .17	− .36, .016	.07
Sample size (MCE)					.005	− .016, .026	.6

	Model R^2^	Mean effect size	N effect sizes included	Homogeneity Q	B	95% CI. LL, UL	p-value
*B Disability*							
Intervention: duration [weeks]	.15	1.7	37	2	.1	− 0.3, 0.95	.3
Intervention: frequency [N_Trainings_/week] data transformed from U-shaped association					.26	− .61, 1.1	.6
Intervention: time per session [minutes] Data transfomed from negative log association					− 1.0	− 3.1, .95	.3
PEDro sum score [points]					− .04	− .37, .30	.8
Sample size (MCE)					− .003	− .06, .06	.9

For each single analysis, effect sizes, number of included effect sizes, homogeneity, the regression coefficient B, its confidence interval (CI) and the corresponding p-value are displayed. Legend: LL, lower level, UL, upper level.

**Figure 2:**
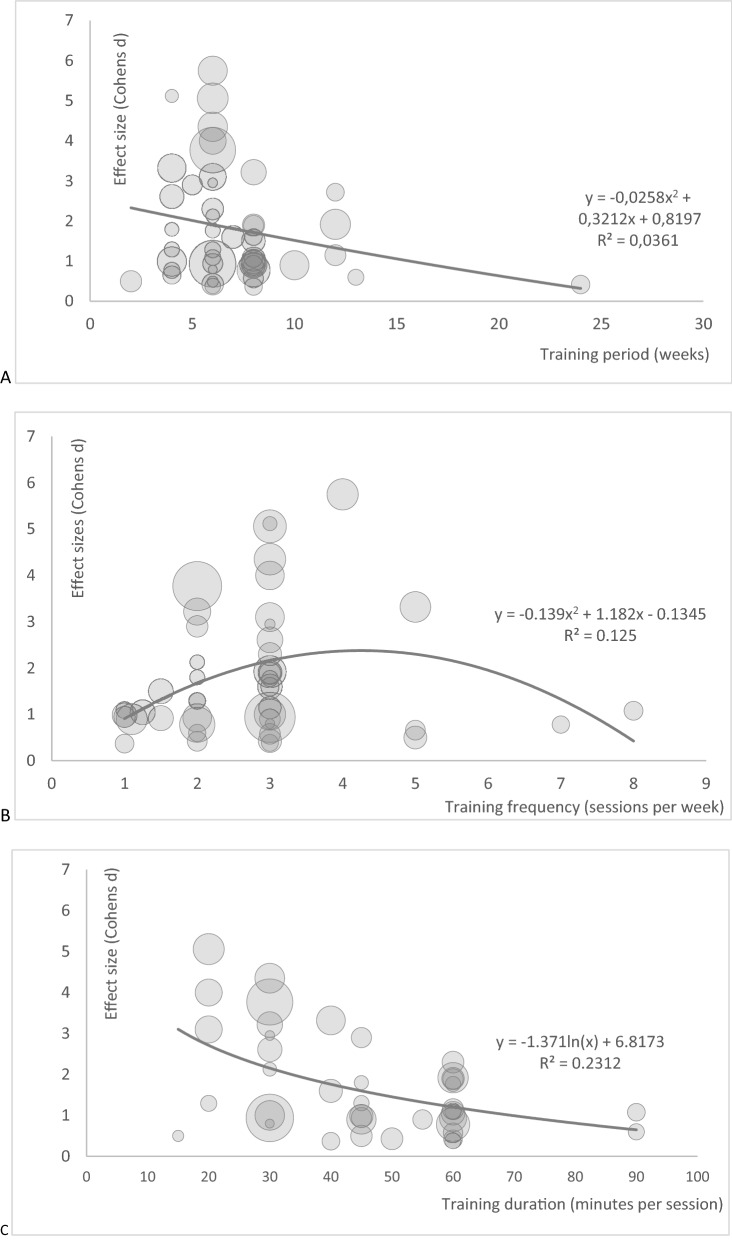
Meta-regression bubble plots for the dependent variable Cohens d (pain), independent variable training period (weeks, A), training frequency (times/week, B) and training duration (minutes, C). The weighting is illustrated by the size of the bubbles.

More detailed information on the meta-regressions are depicted in Fig. 2. The training period showed no systematic impact on the effect size for pain intensity (Fig. 2A). Training frequency showed an inverted U-shaped association with the effect size (13% variance explanation) (Fig. 2B), training duration showed a logarithmic association with the pain effect size (23% variance explanation; Fig. 2C). Low quality evidence suggested that training 3 to 5 times per week leads to the largest effect of stabilisation exercise in chronic, non-specific low back pain patients. Quality of evidence was downgraded due to risk of bias (− 1), downgraded due to imprecise data (wide confidence intervals, − 1), downgraded (− 1) due to (some) uncertainty about directness, and upgraded due to dose-response-relationship (+ 1).

### Risk of bias across studies

**Figure 3:**
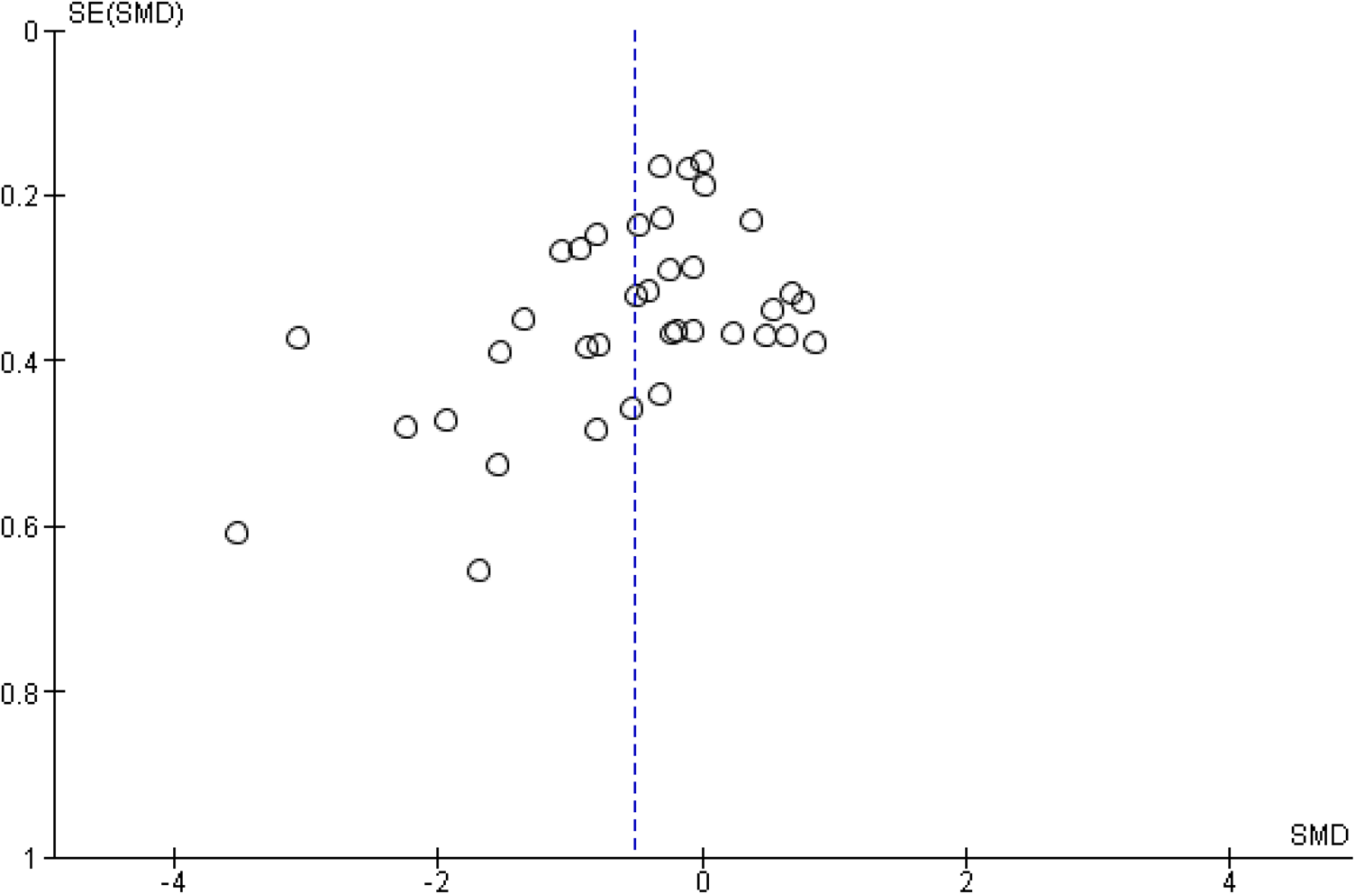
Funnel plot of all studies included. Each first sustainability SMD (standard mean differences and their belonging SE (standard errors) are plotted.

The risk of bias across studies (publication bias) is, by means of a funnel plot, highlighted in Fig. 3. It reveals an unclear, but rather low, risk of publication bias.

## Discussion

This systematic review with meta-regression examined the dose-response-relationship of stabilisation exercise interventions in chronic, non-specific low back pain patients and, thus, derived recommendations for the stabilisation exercises’ training characteristics in this special cohort.

### Summary of main results

The main findings of the presented meta-regression are that: (1) moderate quality evidence indicates that a training duration of 20 to 30 min elicits the largest impact on the effect sizes on both pain and disability of core-specific stabilisation interventions in non-specific chronic low back pain patients, (2) low quality evidence advocates that training 3 to 5 times per week leads to the largest effect of core-specific stabilisation exercise in chronic, non-specific low back pain patients with an inverted U-shaped association with the effect size and (3) no systematic impact of the training period (duration of intervention in weeks) on the effect size for pain intensity was found.

### Comparison with other evidence

Saragiotto et al.^2^ reported a wide range of 20 days to 12 weeks in the period of the applied motor control intervention programmes in their meta-analysis. The number of treatment sessions per week varied from 1 to 5. This partly covers the results of our presented meta-regressions. Nevertheless, a detailed analysis on the effect of training characteristics on pain reduction is missing in their systematic review^2^. The current evidence only proves the use of general and stabilisation exercise (covering sensorimotor, stabilisation and/or core stability) in the therapy of chronic non-specific low back pain^2^. Regarding the training period/duration (weeks of intervention), our results showed that the duration of intervention (in weeks) presented no systematic impact on the effect size for pain intensity. Taking the current knowledge on the effects and adaptation of sensorimotor training into account, a duration of about six weeks seems to be both feasible and effective. This is in accordance with our quantitative results (mean duration of 7.0 ± 3.3 weeks). However, future research is required to define evidence-based recommendations of this aspect.

Low quality evidence supports an inverted U-shaped association of the training frequency (sessions per week) with the effect size on improvement of pain and disability in chronic, non-specific low back pain patients. The overall relationship between (the amount of) physical activity and low back pain is considered to be U-shaped. This means that both the absence of exercise and extremely high levels of physical activity (elite sports) may lead to an increase in the risk of developing (low) back pain. In contrast, a "normal" (medium) level of physical activity shows the lowest risk and, therefore, appears to be protective^2–4,8,9^. In this context, our findings of adopting a dose of 3 to 5 sessions per week covers this. In addition, moderate quality evidence indicates that a training duration of 20 to 30 min elicits the largest impact on the effect sizes on pain and disability; this may correspond to the patients’ essential need of achieving pain reduction with the minimum effort (time). Nevertheless, this is partly in contrast to van Tulder's result^4^. They concluded that exercise interventions with a high dosage (> 20 h) have the highest effect. Van Tulder et al.^4^ fail to point out how this dosage should be applied (duration, frequency). Supported by our findings, it may be more effective to reach this dosage with a high frequency, short bout type of intervention. One of the main reasons of failed treatment success in exercise therapy is the low adherence rate of the patients to their scheduled therapy^4^. Lack of time and long journey times to the therapy centre are commonly cited barriers to regularly participating in therapy sessions^72^. Therefore, patients and physiotherapists are constantly searching for the effective dose-response-relationship that could be reduced to the minimum required. Based on our results, we can recommend exercising for more than 2 sessions per week with a minimum of 20 to 30 min per session. Nevertheless, there is still a need for future research on the minimal dosage in the context of stabilisation exercise interventions for chronic, non-specific low back pain patients.

### Practical relevance and recommendations

The training-dose and effect-response relationship between core-specific stabilisation exercise interventions and pain reduction or disability improvement in chronic, non-specific low back pain patients is of great interest to policy makers, health insurers and clinicians, as well as the persons affected. This review proved the (low to moderate) evidence, that a core-specific stabilisation intervention of 3 to 5 times per week, 20 to 30 min per session, has a positive effect on pain reduction and improvement of disability in low back pain patients. Conclusively, we suggest the following graded recommendations:

Grade A recommendation: At the group level, stabilisation exercise is likely to be most effective to treat non-specific low back pain when it is scheduled with a time per session of 20–30 min.

Grade C recommendation: At the group level, stabilisation exercise to treat non-specific low back pain is potentially most helpful when it is scheduled three to five times a week.

### Future study

Nevertheless, the evidence of more detailed training specifica (training intensity: number of exercises per session, repetitions per exercise, sets per exercise, rest after exercise, etc.) remains unclear. Furthermore, the minimal clinically relevant dosage of core-specific stabilisation interventions in chronic, non-specific low back pain patients remains unclear; this may define a future area of low back pain research as there exists a societal pressure of consistently high low back pain prevalence across all lifespans.

## Limitations

### Limitations at the study and outcome levels

A common limitation in exercise trials is the limited possibility to blind the participants. This limitation is increased by the self-reported assessment of pain and pain-related function.

### Limitations at the review level

We only screened the databases PubMed (Medline), Web of Knowledge and the Cochrane Library. Considering the topic of our review, almost all manuscripts of interest should be found therein^73–75^. However, expanding the search to even more databases, like EMBASE, PEDro, CINAHL; AMED, and CENTRAL may would have led to slightly more hits.

The advantage of meta-regressions are, inter alia, that the interventional effect sizes are compared to each other to find a dose-response-relationship, the effect sizes are thus relativized to each other. The estimates found are valid for the isolated intervention group effects comparisons, given by the meta-regression. The mean effects are, given by the nature of the meta-regression, absolute and not in comparison to a control/comparator. The mean effect sizes (refer to the study description and meta-regressions) are thus not directly comparable to those found in meta-analyses where the effects are calculated in comparison to a control/comparator group.

The funnel plot analysis revealed an unclear, but rather low, risk of publication bias within our review. The findings of our (retrospective) meta-regression should be confirmed prospectively, at best adopting a prospective meta-analysis.

#### Sensitivity of the interventions’ name

The interventions of the studies included into our meta-analysis are defined as stabilization exercise. Motor control exercises are classically defined as core-specific dynamic stabilization exercises with an a priori education on deep trunk muscles activation and/or the control of deep muscles activation during exercising. We only included studies with dynamic/exercise parts. When solely stabilisation exercises without pre-conditioning are performed, they are often called “coordination”, “stabilisation”^5^, “sensorimotor”^76^ or even as well “motor control”^2^ exercise. As described above, the term “motor control exercise” may be slightly too sensitive for the interventions included into our review. In contrary, the terms “sensorimotor”, “coordination” and “stabilisation” training/exercise may be too general. Consequently, we name the intervention “stabilisation exercise” to highlight that the stabilisation/active/dynamic parts of the originally described as “motor control exercise”-theorem are adopted. Nevertheless, the intervention could also be called “motor control stabilization exercise” or “sensorimotor exercise”.

## Conclusions

A training frequency of 3 to 5 times per week (low quality evidence) with a training duration of 20 to 30 min (moderate quality evidence) per session causes the largest impact on the effect sizes (both in pain and disability) of stabilisation exercise in low back pain patients. However, the training period showed no systematic impact on the effect size for pain intensity. Future work is required to enhance the quality of the evidence of our findings, possibly focussing on the definition of a minimum dosage.
